# Analysis and Prediction of Relapse Risk Factors in Community-Dwelling Individuals With Schizophrenia: Retrospective Cohort Study

**DOI:** 10.2196/86991

**Published:** 2026-10-28

**Authors:** Weibo Zhang, Dian Zhu, Xiaolan Wang, Ruilan Li, Hong Jiang, Yanli Liu, Jun Cai, Zhao Liu, Ling Wang

**Affiliations:** 1 Shanghai Institute of Infectious Disease and Biosecurity, Fudan University, No.130, Dong'an Rd., Xuhui Dist., Shanghai, 200032, China, 86 13818782188; 2 Shanghai Mental Health Center, Shanghai Jiao Tong University School of Medicine, Shanghai, China; 3 Minhang District Mental Health Center of Shanghai, Shanghai, Shanghai, China; 4 School of Art, Design and Media, East China University of Science and Technology, Shanghai, China; 5 Department of Maternal, Child and Adolescent Health, School of Public Health, Fudan University, Shanghai, China; 6 School of Design, Shanghai Jiao Tong University, Shanghai, China

**Keywords:** schizophrenia, relapse prediction, community-based rehabilitation, machine learning, explainable AI

## Abstract

**Background:**

Schizophrenia is a severe mental disorder characterized by high disability and relapse rates, posing significant challenges to public safety and community-based mental health management.

**Objective:**

This study aimed to develop and validate a model for identifying hospitalization-related severe relapse risk among individuals with schizophrenia and to determine the key factors associated with this risk.

**Methods:**

A total of 44,804 electronic records of community-managed individuals with schizophrenia were extracted from an information management system between January 1, 2022, and December 31, 2023. The dataset included demographic information, clinical records, interview data, policy support, and management indicators to construct a comprehensive feature system. A Light Gradient Boosting Machine (LightGBM) machine learning algorithm was used for model development, with cost-sensitive learning applied to address class imbalance. Model interpretability was enhanced using Shapley additive explanations (SHAP) to identify the most influential predictors of relapse risk.

**Results:**

After screening, a total of 39,818 community-dwelling individuals with schizophrenia were included in this study, of whom 3717 (9.33%) experienced hospitalization-related severe relapse. The dataset was randomly divided using stratified sampling into a training set (n=31,854, 80%) and an independent test set (n=7964, 20%). Among the 6 machine learning models evaluated, LightGBM showed leading and balanced performance, yielding an area under the receiver operating characteristic curve of 0.95, an area under the precision–recall curve of 0.79, a relapse-class recall of 0.77, a precision of 0.71, and an *F*_1_-score of 0.74 in the independent test set. SHAP-based interpretability analysis identified medication persistence (23.30%), specialist recommendation: medication adherence training (19.74%), number of follow-ups (7.38%), age (3.72%), and educational attainment (3.58%) as the most influential predictors. Higher hospitalization-related severe relapse risk was generally associated with interrupted or refused medication use, more frequent follow-ups, younger age, and higher educational attainment, whereas sustained medication use, better occupational functioning and productive work, greater social participation, and better social functioning were associated with lower predicted risk.

**Conclusions:**

This study developed a hospitalization-related severe relapse risk identification model that integrates multidimensional factors reflecting the clinical and functional complexity of schizophrenia. The findings provide an evidence-based foundation for risk identification, targeted intervention, and the optimization of community mental health services to reduce hospitalization-related severe relapse risk and support long-term recovery.

## Introduction

Schizophrenia is a major global public health concern characterized by multidimensional impairments in perception, cognition, and emotion [1]. According to the Global Burden of Disease (GBD) Study 2019, an estimated 23.6 million people worldwide are affected by schizophrenia [2], with an increasing incidence over time [3]. Its high relapse rate severely compromises functional recovery and poses substantial challenges to long-term disease management, particularly within community-based mental health systems [4-6], further threatening the safety and well-being of families and communities [7].

The early identification and prediction of relapse have long been priorities in schizophrenia research [8-10]. Previous studies have reported that factors such as medication adherence, substance misuse, insight [11], exposure to stressful life events [12], social support, employment status [13], sleep quality [8], and adverse drug reactions [14] are closely associated with relapse [15]. Although electronic health records (EHRs) hold great potential for relapse or rehospitalization prediction, several limitations persist [16]. Relapse is widely recognized as the result of complex interactions among biological, psychological, and social factors, yet systematic investigations within community-based rehabilitation settings remain scarce. In practice, most patients with schizophrenia rely primarily on long-term community follow-up for disease management. However, existing studies often rely on data from single medical institutions with limited sample sizes, restricting their ability to reflect the real-world disease trajectory. Additionally, data collection methods vary across settings, and some variables depend on subjective assessments, which may weaken model generalizability and external validity.

While machine learning techniques offer advantages over traditional statistical methods in handling high-dimensional, complex real-world data and in distinguishing relapse from nonrelapse cases [17], existing models still face limitations in robustness and scalability [13,18,19]. In community mental health systems, predictive models must not only achieve high accuracy but also demonstrate interpretability and operational feasibility, enabling frontline practitioners to derive actionable intervention insights.

To address these gaps, this study developed an information management system (IMS) dataset encompassing community rehabilitation institutions across Shanghai and including 39,818 individuals with schizophrenia. The dataset integrates clinical diagnostic information, demographic characteristics, and community follow-up records, providing a comprehensive representation of relapse-related risk characteristics in real-world community mental health management settings. Furthermore, explainable machine learning techniques were used to improve predictive performance while elucidating the relative contributions of different risk factors to model predictions. The aim of this study was to develop a decision-support tool that can be integrated into community mental health service workflows to support risk identification and timely intervention, thereby providing empirical evidence for the monitoring of hospitalization-related severe relapse risk among individuals with schizophrenia in community settings.

## Methods

### Study Design and Setting

This study was based on the IMS for community-based rehabilitation of patients with schizophrenia, developed and maintained by the Shanghai Mental Health Center; the system continuously monitors and records patients’ rehabilitation data across 16 districts. Patients experiencing acute or severe psychotic episodes were admitted to psychiatric hospitals for inpatient treatment, while those in clinically stable phases were transferred to home-based and community-based rehabilitation programs. Within these community rehabilitation centers, psychiatrists at outpatient clinics were responsible for recording and uploading follow-up information to the IMS. Additionally, family members or other informal caregivers were invited to participate in the evaluation process, providing supplementary observations of patients’ behavior and daily functioning, thereby contributing to the establishment of a unified and comprehensive IMS database.

### Sampling Strategy and Participants

A stratified cluster sampling strategy was adopted to select participants for this study. First, based on the level of urbanization, the 16 administrative districts of Shanghai were categorized into 3 tiers: central urban districts, semicentral suburban districts, and suburban districts, which served as the sampling framework. Following the principle of cluster sampling, Huangpu District and Yangpu District were selected from the central urban tier, Pudong New Area from the semicentral suburban tier, and Baoshan, Minhang, and Jiading Districts from the suburban tier. All individuals with schizophrenia who had follow-up records in the Shanghai Mental Health Information System between January 2022 and December 2023 within the selected districts were included in the initial sample (n=44,804). Participants were then screened according to predefined inclusion and exclusion criteria.

The inclusion criteria were as follows: (1) officially registered in the Shanghai Mental Health IMS; (2) aged 18 years or older; and (3) diagnosed with schizophrenia according to the *International Classification of Diseases, Tenth Revision (ICD-10)* criteria. The exclusion criteria were as follows: deceased individuals, those who had migrated outside Shanghai during the data collection period, long-term inpatients, and individuals younger than 18 years. After applying these criteria, a total of 39,818 patients were retained for subsequent modeling and analysis.

During feature selection, personal identifiers (eg, patient name, hospital name, and rehabilitation site), time-dependent variables irrelevant to the prediction task (eg, follow-up date and record creation time), and potential data leakage variables (eg, residential address and name of attending psychiatrist) were excluded. Each patient record contained structured information, including demographic characteristics, clinician-rated diagnostic data, social interaction indicators, and treatment-related management details. Additionally, unstructured text fields documented behavioral observations, participation in rehabilitation programs, and individualized recommendations from psychiatric specialists.

In this study, relapse was operationally defined as a patient’s transfer from community-based rehabilitation to a psychiatric hospital for inpatient treatment due to acute exacerbation or deterioration of psychotic symptoms [20]. Hospitalizations related to physical comorbidities or nonpsychiatric causes were excluded. Only hospital admissions that psychiatrists determined to be acute psychotic relapse events were classified as relapse cases. If such a transfer occurred during the observation period, the patient was labeled as a positive (relapse) case; otherwise, the patient was classified as a negative (nonrelapse) case.

For relapse cases, data from the last follow-up prior to hospitalization were used as predictive variables for subsequent relapse outcomes. For nonrelapse cases, data from the most recent follow-up were selected as predictive variables, and the absence of relapse during the subsequent observation period was used as the outcome label. This approach ensured temporal precedence of predictors relative to outcomes, thereby preventing bias from potential data leakage.

Because the original IMS database could contain multiple follow-up records for the same patient, the analysis dataset was constructed at the patient level before model development. Each patient was restricted to 1 predefined index record. For patients who experienced hospitalization-related severe relapse, the index record was defined as the last community follow-up record before hospitalization due to acute exacerbation or deterioration of psychotic symptoms. For patients without hospitalization-related severe relapse during the observation period, the index record was defined as the last valid community follow-up record during the observation period. After this procedure, each patient contributed only one record to the final analysis dataset.

### Feature Construction

The initial feature pool comprised 52 candidate variables, spanning five thematic domains: (1) sociodemographic characteristics; (2) clinical diagnosis and medical history; (3) medication management and follow-up; (4) rehabilitation participation; and (5) rehabilitation interventions and specialist recommendations. During data preprocessing, variables with more than 50% missing values were excluded [21]. Prior to feature selection, a variance thresholding approach was applied to remove variables with negligible predictive utility. Specifically, features with zero variance and near-zero variance (ie, nonzero occurrence rate below 0.1%) were eliminated. After preprocessing, 41 features were retained for model training (Table 1). These features were subsequently organized into 5 major domains (Multimedia Appendix 1), as summarized in Table 1.

**Table 1.** Summary of feature categories.

Domain	Variables
(1) Sociodemographic characteristics	Age; sex; educational attainment; marital status; long-term bedridden status or loss of mobility; family supervision status; participation in community rehabilitation services; number of follow-ups
(2) Clinical diagnosis and medical history	Antipsychotic treatment status; reason for untreated status; recent stressful events; social functioning assessment; insight into illness; history of violent or disruptive behavior; mode of illness onset; past medical history; disability severity level
(3) Medication management and follow-up	Medication persistence; medication administration pattern; medication adherence; timeliness of clinical follow-up
(4) Rehabilitation participation	Social participation; sleep quality; dietary status; personal self-care ability; household functioning; occupational functioning and productive work; acquisition of new knowledge and skills; interpersonal social interaction
(5) Rehabilitation interventions and specialist recommendations	Rehabilitation intervention (daily living and vocational skills); rehabilitation intervention (vocational training); rehabilitation intervention (learning ability); rehabilitation intervention (social interaction); other rehabilitation interventions; specialist recommendation: treatment adjustment; specialist recommendation: vocational rehabilitation training; specialist recommendation: symptom monitoring training; specialist recommendation: learning skills training; specialist recommendation: social skills training; specialist recommendation: daily living skills training; specialist recommendation: medication adherence training

### Ethical Considerations

This study was based on the analysis of routinely collected community follow-up data, with all personally identifiable information removed prior to analysis. The Ethics Committee of the Shanghai Mental Health Center approved this study (institutional review board registration number: IORG0002202; Federalwide Assurance number: FWA00003065).

### Statistical Analysis and Model Training

#### Statistical Analysis

Baseline statistical analyses were performed using SPSS Statistics software (version 26.0; IBM Corp). For categorical variables, chi-square tests were conducted to examine group differences in variable distributions across relapse statuses. For multiple-response categorical variables, multiple-response analyses were applied to calculate the frequency of each response option, followed by between-group comparisons. For continuous variables with skewed distributions or those violating the assumption of normality, nonparametric tests were used. Specifically, independent-samples *t*-tests or Mann-Whitney *U* tests were used for comparisons between 2 groups, whereas Kruskal-Wallis H tests were used for comparisons involving more than 2 groups. All statistical tests were 2-tailed, and a *P* value <.05 was considered statistically significant.

#### Model Training

In the present study, the original dataset exhibited severe class imbalance, with a ratio of approximately 10:1 between the majority (nonrelapse) and minority (relapse) classes. Such an extreme imbalance can cause conventional classifiers to exhibit strong bias toward the majority class, thereby achieving deceptively high overall accuracy but extremely poor recall for the minority class—resulting in suboptimal identification of relapse cases and limited generalizability.

A stratified random sampling strategy was applied to split the dataset into a training set (31,854/39,818, 80%) and an independent test set (7964/39,818, 20.0%), ensuring that the relapse-to-nonrelapse ratio remained consistent across both subsets. All feature selection and hyperparameter optimization procedures were strictly confined to the training set to prevent information leakage. Final model performance was evaluated exclusively on the held-out test set to ensure an unbiased assessment [22]. Class imbalance was addressed using cost-sensitive learning. For tree-based gradient boosting models (Light Gradient Boosting Machine [LightGBM] and extreme gradient boosting [XGBoost]), scale_pos_weight was applied. For random forest (RF), support vector machine (SVM), and logistic regression (LR), class_weight was used. For adaptive boosting (AdaBoost), sample weights (sample_weight) were assigned. Higher misclassification costs were assigned to the minority class (relapse), and all weighting parameters were jointly optimized with other hyperparameters through 5-fold stratified cross-validation and grid search. This strategy improves minority-class sensitivity without altering the true class distribution of the test set.

Missing-value handling was implemented according to model capabilities. LightGBM and XGBoost support native missing-value processing by learning an optimal routing direction for missing observations at each tree split; therefore, original missing values were retained without imputation. For the 4 models that do not support native missing values (RF, AdaBoost, SVM, and LR), median imputation was performed using statistics estimated exclusively from the training set. For SVM and LR, feature standardization was subsequently applied, with the scaler fitted only on the training set and then applied to the test set, thereby preventing any information leakage.

A total of 14 variables contained missing values, with missingness rates ranging from 0.6% to 17.2%. Under this framework, all samples retained their natural missing-data patterns and were handled through the native mechanisms of tree-based models whenever possible. Finally, model-derived feature importance was examined as part of the model interpretation process, and Shapley additive explanations (SHAP) analysis was subsequently used to quantify feature contributions to the final LightGBM model [23].

#### Exploratory Temporal Robustness Check

An exploratory temporal robustness check was conducted according to the calendar year of the retained patient-level index record. Using the same modeling pipeline as in the primary random 80/20 split analysis, the LightGBM model was trained on the 2022 index-record cohort and evaluated on the 2023 index-record cohort. Because the calendar year of the retained index record was partly influenced by the index-record selection rule, this analysis was prespecified as an exploratory robustness check rather than as a conventional independent temporal validation. For patients without hospitalization-related severe relapse, the retained index record was the last valid follow-up record during the observation period, whereas for relapse cases, the retained index record was the last follow-up before hospitalization. Therefore, the calendar-year split was not fully independent of outcome status.

#### Model Implementation and Optimization

The machine learning models were implemented in Python 3.10 using LightGBM (version 3.3.5). In this study, 6 widely used machine learning algorithms were evaluated, including LightGBM, RF, AdaBoost, SVM, LR, and XGBoost.

Hyperparameter optimization was performed using GridSearchCV with 5-fold stratified cross-validation, using area under the receiver operating characteristic curve (ROC-AUC) as the optimization metric. Class imbalance was addressed through cost-sensitive learning rather than resampling. Specifically, gradient boosting models (LightGBM and XGBoost) used the scale_pos_weight parameter, while RF, SVM, and LR used class_weight, and AdaBoost incorporated class-dependent sample weights (sample_weight). The hyperparameter search spaces and the corresponding optimal configurations for all models are provided in Multimedia Appendix 2.

#### SHAP Analysis

To enhance the clinical interpretability of the model, SHAP analysis was performed on the final trained LightGBM model to quantify the direction and magnitude of each feature’s contribution to the predicted outcome.

SHAP values are derived from Shapley values in cooperative game theory and provide both local explanations at the individual patient level and global feature importance at the cohort level.

In the SHAP summary plot, each point represents an individual patient. The x-axis indicates the SHAP value, which reflects the contribution of a given feature to the model output. Positive SHAP values indicate an increased predicted risk of relapse, whereas negative values indicate a lower predicted risk. Features are ranked according to their mean absolute SHAP values, with those at the top of the plot exerting a greater overall influence on model predictions.

## Results

### Baseline Characteristics of the Included Patients

The baseline characteristics of community-dwelling patients with schizophrenia showed that the median age was 60 (IQR 47‐69) years, and 53.2% (21,170/39,818) of the participants were female. Among the 39,818 individuals included in this study, 3717 (9.3%) experienced relapse, while 36,101 (90.7%) did not.

Between-group comparisons indicated that patients in the relapse group were significantly younger than those in the nonrelapse group (z=−20.78, *P*<.001). Regarding educational attainment, a significantly higher proportion of patients with a college education or above was observed in the relapse group (839/3717, 22.6%) compared with the nonrelapse group (*χ*²_2_=98.73, *P*<.001). In terms of marital status, the proportion of unmarried patients was higher in the relapse group (2054/3717, 55.3%) than in the nonrelapse group (15,234/36,101, 42.2%; *χ*²_2_=339.34, *P*<.001; Table 2). The detailed data provided in Multimedia Appendix 1.

**Table 2.** Demographic and baseline characteristics of patients by relapse status.

Variable	Nonrelapse (n=36,101)	Relapse (n=3717)	Total (N=39,818)	Statistic	*P* value
Age, median (IQR)	61 (47-69)	54 (42-64)	60 (47-69)	–20.78^a^	<.001^b^
Number of follow-ups, median (IQR)	5 (3-7)	5 (2-10)	5 (3-8)	–0.06^a^	.95
Sex, n (%)				48.25 (2)^c^	<.001^b^
Male	16,706 (46.3)	1942 (52.2)	18,648 (46.8)		
Female	19,395 (53.7)	1775 (47.8)	21,170 (53.2)		
Educational attainment, n (%)				98.73 (2)^c^	<.001^b^
Primary school or illiterate/semi-illiterate	4012 (11.1)	264 (7.1)	4276 (10.7)		
Junior high school/senior high school	25,763 (71.4)	2614 (70.3)	28,377 (71.3)		
Junior college/bachelor’s degree/master’s degree or above	6326 (17.5)	839 (22.6)	7165 (18)		
Marital status, n (%)				339.34 (2)^c^	<.001^b^
Unmarried	15,234 (42.2)	2054 (55.3)	17,288 (43.4)		
Married	16,488 (45.7)	1114 (30)	17,602 (44.2)		
Other	4379 (12.1)	549 (14.8)	4928 (12.4)		
Long-term bedridden status or loss of mobility, n (%)				0.11 (2)^c^	.74
No	35,105 (97.2)	3611 (97.1)	38,716 (97.2)		
Yes	996 (2.8)	106 (2.9)	1102 (2.8)		
Family supervision status, n (%)				276.73 (2)^c^	<.001^b^
No supervision	199 (0.6)	40 (1.1)	239 (0.6)		
Weak	4076 (11.3)	755 (20.3)	4831 (12.1)		
Good	31,826 (88.2)	2922 (78.6)	34,748 (87.3)		
Antipsychotic treatment status, n (%)				2.01 (2)^c^	.16
No	269 (0.7)	20 (0.5)	289 (0.7)		
Yes	35,832 (99.3)	3697 (99.5)	39,529 (99.3)		

a *z* test values.

b *P*<.05 indicates statistical significance.

c Chi-square test (*df*).

### Model Development and Performance

To systematically evaluate risk factors associated with relapse in schizophrenia and their relative importance, multiple machine learning models were constructed and compared, including LightGBM, AdaBoost, XGBoost, SVM, LR, and RF.

Given that nonrelapse cases were dominant in the dataset, relying solely on overall accuracy and weighted metrics (weighted recall, weighted precision, and weighted *F*_1_-score) may be biased toward the majority class. Therefore, this study additionally focused on relapse-class-specific performance and macroaveraged metrics (macro recall, macro precision, and macro *F*_1_-score) to more objectively evaluate model performance under class imbalance conditions.

Among the 6 models evaluated, the 2 gradient boosting models (LightGBM and XGBoost) achieved the best overall performance, both attaining ROC-AUC values of approximately 0.95 and substantially outperforming LR (ROC-AUC=0.85). These findings suggest that boosting-based models are more capable of capturing the complex nonlinear relationships and high-dimensional interactions present in community mental health EHRs (Figure 1). LightGBM demonstrated the best overall performance, achieving an ROC-AUC of 0.95, an area under the precision–recall curve (PR-AUC) of 0.79, a relapse-class recall of 0.77, a precision of 0.71, and an *F*_1_-score of 0.74. The optimal hyperparameter configuration was learning_rate=0.1, max_depth=13, n_estimators=100, num_leaves=31, and scale_pos_weight=3.

**Figure 1.**
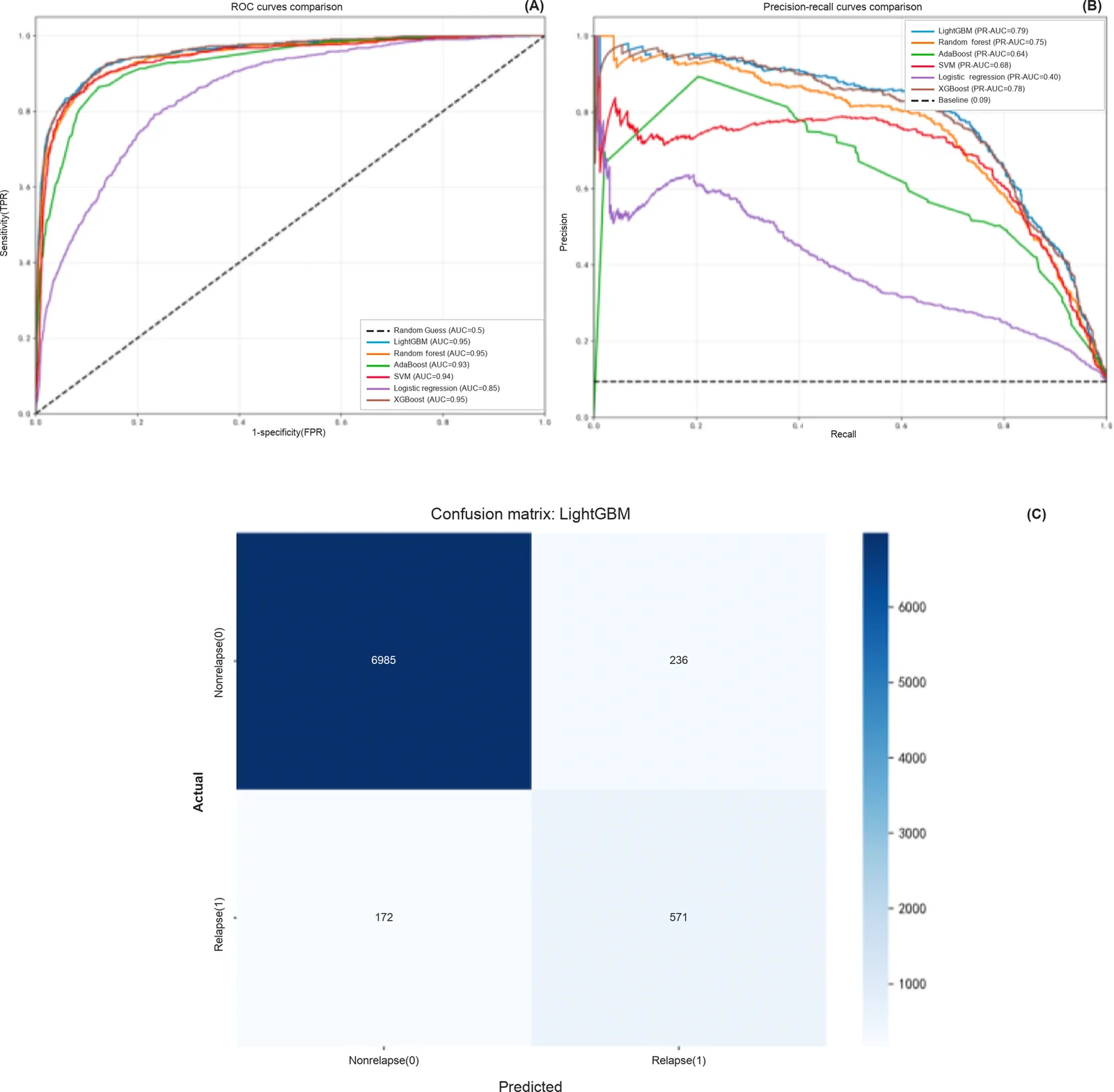
Performance evaluation of the hospitalization-related severe relapse risk identification model. (A) Receiver operating characteristic curve illustrating the model’s overall ability to discriminate between relapse and nonrelapse patients. (B) Precision-recall curve assessing the model’s performance in identifying the minority class (relapse patients), particularly in terms of precision and recall. (C) Confusion matrix of the Light Gradient Boosting Machine (LightGBM) model, showing the number of correctly classified nonrelapse (stable) and relapse patients. AdaBoost: adaptive boosting; FPR: false positive rate; SVM: support vector machine; TPR: true positive rate; XGBoost: extreme gradient boosting.

Notably, the models exhibited a clear sensitivity–precision trade-off in identifying relapse cases. AdaBoost and SVM achieved relatively high relapse-class recall values of 0.80 and 0.82, respectively, indicating greater sensitivity in detecting relapse cases. However, their precision values were comparatively low, at 0.49 and 0.56, respectively, suggesting a higher number of false-positive predictions. XGBoost showed overall performance highly comparable to that of LightGBM, with similar accuracy, macro *F*_1_-score, relapse-class recall, relapse-class precision, relapse-class *F*_1_-score, and ROC-AUC (Table 3). However, the PR-AUC of XGBoost was slightly lower than that of LightGBM. Considering that PR-AUC is particularly informative under class imbalance, and that LightGBM maintained a favorable balance between relapse-class recall and precision, LightGBM was retained as the primary model for subsequent interpretability and sensitivity analyses.

**Table 3.** Performance of different machine learning models.

Model	Accuracy	Weighted_recall	Weighted_precision	Weighted_*F*_1_	Macro_recall	Macro_precision	Macro_*F*_1_	Relapse_recall	Relapse_precision	Relapse_*F*_1_	Nonrelapse_Recall	Nonrelapse_Precision	Nonrelapse_*F*_1_	ROC_AUC^a^	PR_AUC^b^
LightGBM^c^	0.95	0.95	0.95	0.95	0.87	0.84	0.85	0.77	0.71	0.74	0.97	0.98	0.97	0.95	0.79
RF^d^	0.94	0.94	0.94	0.94	0.85	0.82	0.83	0.73	0.68	0.70	0.96	0.97	0.97	0.95	0.75
AdaBoost^e^	0.90	0.90	0.93	0.91	0.86	0.74	0.78	0.80	0.49	0.61	0.92	0.98	0.95	0.93	0.64
SVM^f^	0.92	0.92	0.94	0.93	0.88	0.77	0.81	0.82	0.56	0.67	0.93	0.98	0.96	0.94	0.68
LR^g^	0.77	0.77	0.91	0.82	0.78	0.62	0.63	0.78	0.26	0.39	0.77	0.97	0.86	0.85	0.40
XGBoost^h^	0.95	0.95	0.95	0.95	0.86	0.84	0.85	0.76	0.71	0.74	0.97	0.97	0.97	0.95	0.78

a ROC-AUC: area under the receiver operating characteristic curve.

b PR-AUC: area under the precision–recall curve.

c LightGBM: Light Gradient Boosting Machine.

d RF: random forest.

e AdaBoost: adaptive boosting.

f SVM: support vector machine.

g LR: logistic regression.

h XGBoost: extreme gradient boosting.

In addition to discrimination performance, model calibration was evaluated using calibration curves and Brier scores. The unweighted baseline model achieved a Brier score of 0.036, indicating good agreement between predicted probabilities and observed outcomes. The cost-sensitive LightGBM model yielded a slightly higher Brier score of 0.040. Although cost-sensitive learning improved relapse detection performance, a modest reduction in calibration was observed, reflecting the inherent trade-off between sensitivity and probability calibration (Multimedia Appendix 2).

### Model Interpretability

The SHAP-based interpretability analysis identified the key predictors contributing to model predictions, ranked according to their relative importance (Figure 2). Among all features, medication persistence contributed the most to model prediction, accounting for 23.30% of the total feature importance, suggesting that medication-management information was an important predictive signal. The next most influential predictors were specialist recommendation: medication adherence training (19.74%), number of follow-ups (7.38%), and age (3.72%), indicating that these variables were closely related to the relapse risk patterns captured by the model. Other important predictors included educational attainment (3.58%), occupational functioning and productive work (3.11%), family supervision status (2.94%), and social functioning assessment (2.80%). The SHAP summary plot further showed the direction of associations between these features and model-predicted risk. Overall, refusal of medication, intermittent medication use, or poorer medication persistence during disease management, as well as a greater number of follow-ups, were generally associated with higher model-predicted risk. Although the number of follow-ups did not show a significant univariate between-group difference in the baseline comparison, its SHAP contribution reflects its conditional contribution to model prediction after accounting for other clinical, medication-management, and functional variables. In addition, younger age and higher educational attainment were generally associated with higher predicted risk. Poorer occupational functioning and productive work, specialist recommendation: treatment adjustment, higher disability severity level, and lower social participation were also associated with increased predicted risk.

**Figure 2.**
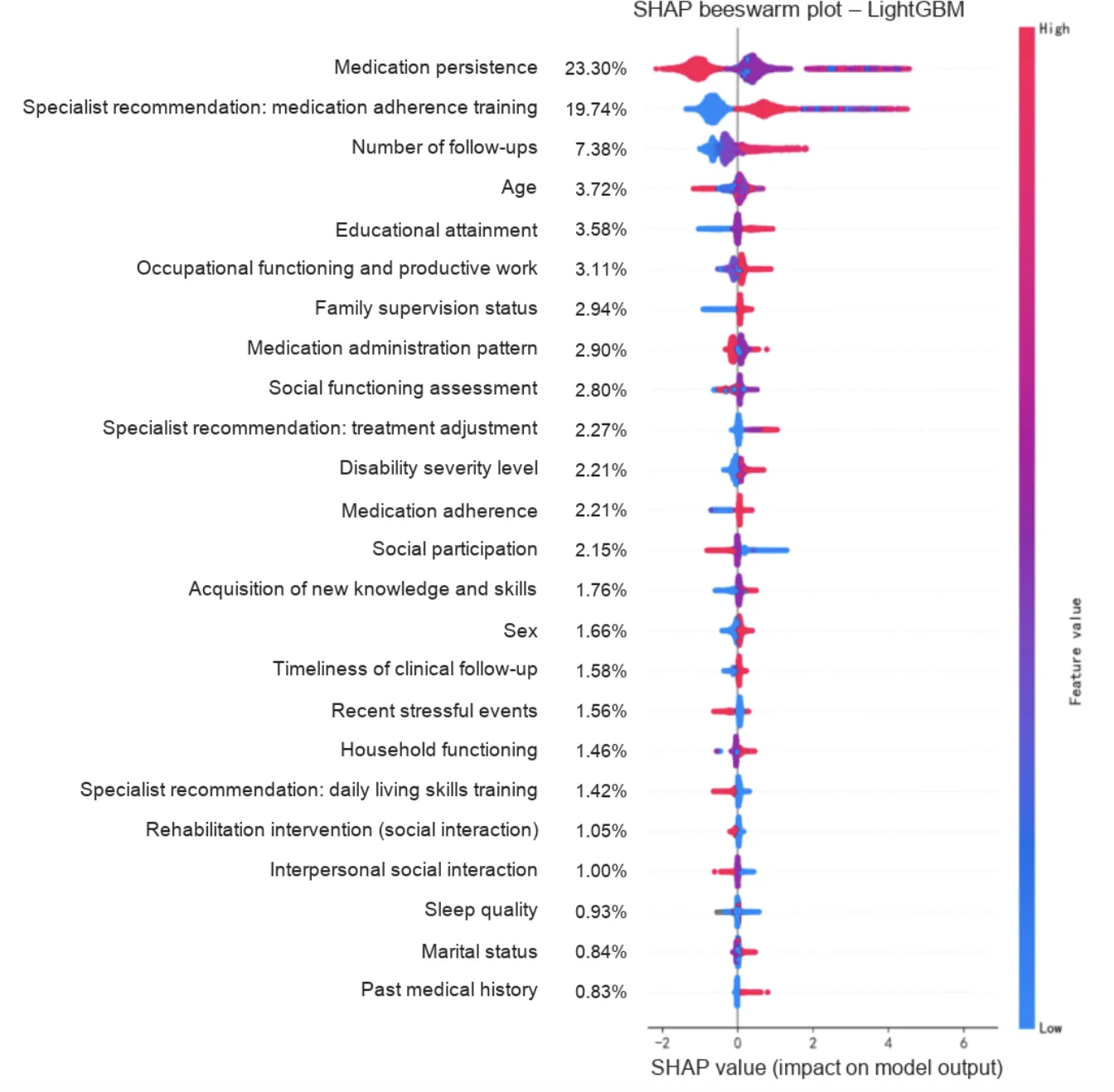
Shapley additive explanations values for hospitalization-related severe relapse risk identification, where blue and red indicate low and high feature values, respectively. LightGBM: Light Gradient Boosting Machine.

### Exploratory Temporal Robustness Check

In the exploratory temporal robustness check, the LightGBM model was trained on the 2022 index-record cohort and evaluated on the 2023 index-record cohort using the same modeling pipeline as in the primary random 80/20 split analysis. The 2022 index-record cohort included 9028 patients, with a hospitalization-related severe relapse prevalence of approximately 19.7%. The 2023 index-record cohort included 30,790 patients, with a relapse prevalence of 6.3%. Among the 30,790 patients in the 2023 cohort, 21,566 had retained index records in the fourth quarter of 2023.

The model achieved a ROC-AUC of 0.93 and a PR-AUC of 0.62 in the 2023 index-record cohort, compared with 0.95 and 0.79, respectively, in the primary random 80/20 split analysis. At the default threshold of 0.50, relapse-class recall, precision, and *F*_1_-score were 0.56, 0.63, and 0.60, respectively. When the decision threshold selected from the 2022 training cohort was applied, relapse-class recall increased to 0.61, with a precision of 0.60 and an *F*_1_-score of 0.61.

These results suggest that the model retained discrimination when applied to records indexed in the subsequent calendar year. However, because the calendar year of the retained index record was partly influenced by the index-record selection rule, this analysis should be interpreted as an exploratory temporal robustness check (Table 4) rather than as evidence of conventional independent temporal validation. The lower PR-AUC and threshold-dependent performance in the 2023 cohort may partly reflect differences in relapse prevalence, case mix, and the index-record selection rule. Further details on quarter-wise performance and relapse prevalence are provided in Multimedia Appendix 2.

**Table 4.** Comparison of model performance between the primary random split analysis and the exploratory temporal robustness check.

Data split strategy	ROC-AUC^a^	PR-AUC^b^	Relapse recall	Relapse precision	Relapse *F*_1_-score	Threshold
Primary analysis (random 80/20 split)	0.95	0.79	0.77	0.71	0.74	0.50
Exploratory temporal robustness check (trained on the 2022 index-record cohort and evaluated on the 2023 index-record cohort)	0.93	0.62	0.56	0.63	0.60	0.50
Exploratory temporal robustness check (trained on 2022 cohort, tested on 2023 cohort; threshold selected from training set)	0.93	0.62	0.61	0.60	0.61	0.34

a ROC-AUC: area under the receiver operating characteristic curve.

b PR-AUC: area under the precision–recall curve.

## Discussion

### Main Findings

Using large-scale, real-world community rehabilitation data from 39,818 individuals with schizophrenia recorded in the IMS, this study developed a hospitalization-related severe relapse risk identification model for community mental health management settings. The primary purpose of the model is not to provide precise predictions of relapse within a predefined future time window. Rather, it aims to use information routinely collected during community follow-up visits to dynamically identify patients who may have entered a high-risk trajectory and are more likely to require hospitalization-related intervention. Therefore, the model is intended to support risk screening, risk stratification, and early intervention within community mental health services.

The model integrated multidimensional information, including behavioral characteristics, functional status, interpersonal relationships, and community support, and used LightGBM together with SHAP-based interpretability analysis to identify key risk factors. The results indicated that medication persistence, specialist recommendation: medication adherence training, number of follow-ups, and age were among the most influential predictors of hospitalization-related severe relapse risk. In addition, educational attainment, occupational functioning and productive work, and social functioning assessment also demonstrated substantial importance, suggesting that functional recovery and social adaptation are closely associated with the risk patterns identified by the model [24].

Global feature importance analysis further highlighted the central role of medication management–related factors. Among them, medication persistence and specialist recommendation: medication adherence training were the 2 most influential predictors, while medication adherence also ranked among the key features. SHAP distribution plots showed that sustained medication use and good medication adherence were generally associated with lower risk, whereas intermittent medication use or refusal to take medication was more frequently associated with higher risk. These findings suggest that medication management–related factors play a critical role in the identification of hospitalization-related severe relapse risk. Previous studies have widely recognized medication nonadherence as one of the most important predictors of schizophrenia relapse [15,25], and medication education and supervision are typically associated with improved adherence and lower relapse rates [14,24]. Overall, the findings of this study are generally consistent with existing evidence. However, it is noteworthy that the proportion of patients recorded as having “regular medication adherence” was higher in the relapse group than in the nonrelapse group. For patients with more severe illness or higher relapse risk, community physicians and family members may implement stricter supervision and management, making these patients more likely to be documented as having “regular medication adherence” in follow-up records. In addition, this study found that better occupational functioning and productive work, better social functioning assessment, and better social participation were associated with lower model-predicted risk. This finding is consistent with previous research on social skills training, which has shown that social skills training and functional rehabilitation are typically associated with better social functioning, self-efficacy, and clinical stability [26,27]. Furthermore, Chen et al [28] found that higher levels of social participation are generally associated with lower levels of psychosocial stress and fewer social withdrawal behaviors [29,30].

### Other Findings

Some findings in this study were inconsistent with previous literature. Higher educational attainment was associated with higher model-predicted relapse risk. One possible explanation is that patients with higher education levels may exhibit greater sensitivity to diagnostic labeling and social stigma, which may in turn be reflected in different illness perceptions and treatment attitudes during long-term management. This finding suggests that, in community rehabilitation settings, in addition to symptom control, greater attention should be paid to psychological adaptation, illness perception, and social support among higher-functioning patients. Future longitudinal studies are warranted to further explore the relationship between educational level, social functioning, and long-term disease management [31]. Higher disability severity was associated with higher model-predicted risk. This finding may be explained by patients’ current functional status, including impairments in self-care and social participation. Such functional impairments may increase perceived stigma and reduce self-esteem, thereby increasing patients’ vulnerability to relapse [32].

The present study also observed that younger age was associated with higher model-predicted relapse risk. Consistent with previous studies, younger patients or those with early-onset schizophrenia tend to have higher relapse rates and poorer long-term outcomes. On one hand, patients in the early stages of the illness often show more pronounced symptom instability and lower treatment stability. On the other hand, younger patients more frequently experience treatment discontinuation, loss to follow-up, and less stabilized social functioning. These characteristics have also been associated with higher relapse risk in previous research. In addition, younger patients are often in the process of establishing social roles, and greater pressures related to education, employment, and social adaptation may further complicate disease management [33].

Finally, a higher number of follow-ups was associated with higher model-predicted relapse risk. This finding may reflect the “high-risk intensive follow-up” pattern in community mental health management, whereby patients with unstable conditions, a frequent relapse history, or higher clinical risk receive more intensive community follow-up and health management. Therefore, higher follow-up frequency may reflect not only health care usage intensity but also indirectly indicate underlying disease severity and relapse risk.

Overall, dynamic management information within community mental health service systems may provide important complementary signals for relapse risk identification, further highlighting the potential use of machine learning models based on community EHRs in chronic mental disorder management.

### Model Performance and Clinical Utility

In this study, LightGBM showed leading and balanced performance among the 6 evaluated models, with an ROC-AUC of 0.95, a PR-AUC of 0.79, a relapse-class recall of 0.77, a precision of 0.71, and an *F*_1_-score of 0.74 on the independent test set. These results suggest that the model had good discriminative ability and performed reasonably well in identifying patients at risk of hospitalization-related severe relapse under a highly imbalanced outcome distribution. However, model performance should not be interpreted solely on the basis of ROC-AUC or overall accuracy. In community mental health management, the balance between sensitivity and precision is particularly important. A relapse-class recall of 0.77 indicates that the model identified approximately 77% of hospitalization-related severe relapse cases, while approximately 23% of such cases were not detected. Meanwhile, a precision of 0.71 suggests that most patients classified as high risk subsequently experienced hospitalization-related severe relapse, although false-positive alerts remained present. This trade-off has practical implications for community-based risk management. Lowering the decision threshold may improve the detection of high-risk patients, but it would also increase false-positive alerts, leading to additional follow-up visits, referral assessments, and intervention-related resource use. Conversely, using a stricter threshold may improve precision and reduce unnecessary alerts, but at the cost of missing more patients who may later require hospitalization-related intervention. Therefore, the model is better understood as a risk stratification and decision-support tool rather than a standalone screening instrument or a replacement for clinical judgment.

In community practice, the predicted risk score may be used to support follow-up prioritization and resource allocation. For example, patients with higher predicted risk may be considered for more frequent community follow-up, timely psychiatric reassessment, multidisciplinary review, or individualized management strategies addressing medication management, social functioning, family supervision, and rehabilitation participation. However, the model should not be used to automatically determine treatment adjustment, referral, or hospitalization. Final decisions should remain clinician-led and should integrate the predicted risk score with clinical assessment, patient history, family support, current mental state, and local service resources.

In addition, the slightly higher Brier score observed in the cost-sensitive LightGBM model indicates that improved relapse detection may be accompanied by a modest reduction in probability calibration. Thus, the predicted scores should be interpreted primarily as relative risk indicators for prioritizing community management, rather than as fully calibrated individual probabilities. Future studies should further optimize decision thresholds according to specific service scenarios, available community resources, and an acceptable false-positive burden, and should evaluate calibration and clinical utility before broader implementation.

The exploratory temporal robustness check further suggests that the model retained good discriminative performance when applied to a future cohort within the same community mental health management system. However, temporal robustness should be distinguished from external generalizability. Because all data in this study were derived from the Shanghai community mental health management system, model performance may still be influenced by local follow-up protocols, hospitalization referral criteria, documentation practices, patient characteristics, and community rehabilitation resources. Before broader implementation, the model should be externally validated using independent datasets from different geographic regions, health care systems, and community mental health service settings.

Finally, if the model is integrated into routine community mental health practice, its performance and calibration should be periodically monitored. As community management practices, follow-up routines, patient composition, service resources, and relapse prevalence evolve over time, recalibration or model updating may be required to maintain the model’s long-term applicability and clinical decision-support value.

### Limitations

Several limitations should be acknowledged. First, this study did not use a fully standardized prediction window. For relapse cases, predictors were extracted from the last follow-up assessment before hospitalization; therefore, the model should be interpreted as a tool for identifying hospitalization-related severe relapse risk in real-world community management, rather than as a prognostic model for predicting relapse within a fixed future time horizon. Future studies should incorporate predefined prediction windows, longitudinal sequential data, and time-to-event modeling to evaluate earlier relapse risk detection. Second, the outcome was defined as hospitalization-related severe relapse, namely, psychiatric hospitalization due to acute exacerbation or deterioration of psychotic symptoms. This definition provides an objective and clinically meaningful end point, but it may not capture milder symptom exacerbations managed through outpatient care, medication adjustment, or intensified community follow-up. Third, generalizability remains limited because all data were derived from the Shanghai community mental health management system. Although the exploratory temporal robustness check using the 2022 and 2023 index-record cohorts suggested temporal robustness, the calendar year of the retained index record was partly influenced by outcome status because the index record was selected differently for nonrelapse and relapse patients. Model performance may also be affected by local follow-up protocols, hospitalization referral criteria, documentation practices, and related contextual factors. Future studies should prospectively validate the model in independently assembled cohorts or external datasets and consider periodic recalibration before broader implementation. In addition, some predictors, including specialist recommendations, medication-management variables, and follow-up-related variables, may partly reflect community management intensity or clinicians’ responses to perceived instability rather than patient-level risk factors alone. These variables should therefore be interpreted cautiously. The main analysis used integer encoding for categorical variables, which may impose artificial ordering on nominal variables, particularly for distance-based and linear comparator models. Future studies should consider one-hot encoding or model-native categorical feature handling to minimize the potential influence of encoding choices. Finally, several optimal hyperparameters were located at the boundary of the predefined search grid, suggesting that the search space may not have fully covered all potentially optimal parameter combinations. Therefore, the performance of each model should be interpreted as the performance achieved under the predefined tuning protocol rather than as the theoretical upper-bound performance of the algorithm. Future studies may use broader search ranges, random search, or Bayesian optimization for more exhaustive hyperparameter optimization.

### Conclusions

Based on IMS data for community mental health services, this study developed a hospitalization-related severe relapse risk identification model for individuals with schizophrenia and used LightGBM in combination with SHAP analysis to enhance model interpretability. The study integrated multidimensional variables related to patient behavioral characteristics, social functioning, community management, and rehabilitation, and analyzed community follow-up records from 39,818 patients. The results showed that medication persistence, educational attainment, specialist recommendation for medication adherence training, age, and follow-up management were among the most influential predictors in the model. Specifically, sustained medication use and better occupational functioning and productive work were generally associated with lower predicted relapse risk, whereas higher educational attainment, high disability level, and younger age were more frequently associated with higher predicted relapse risk. Among the machine learning models evaluated, LightGBM demonstrated the leading and balanced performance in identifying relapse cases, suggesting that EHR-based machine learning approaches may provide a promising tool for the identification of hospitalization-related severe relapse risk in community mental health management settings.

## Supplementary material

10.2196/86991 Multimedia Appendix 1 Baseline characteristics, variable coding, and statistical comparisons by relapse status.

10.2196/86991 Multimedia Appendix 2 Detailed machine learning model development, hyperparameter optimization, model validation, sensitivity analyses, and Shapley Additive Explanations feature importance.

## Abbreviations

AdaBoost: adaptive boosting
EHR: electronic health record
GBD: Global Burden of Disease
*ICD-10*: *International Classification of Diseases, Tenth Revision*
IMS: information management system
LightGBM: Light Gradient Boosting Machine
LR: logistic regression
PR-AUC: area under the precision–recall curve
RF: random forest
ROC-AUC: area under the receiver operating characteristic curve
SHAP: Shapley additive explanations
SVM: support vector machine
XGBoost: extreme gradient boosting

## References

[1] Schizophrenia. World Health Organization.

[2] Solmi M, Seitidis G, Mavridis D (2023). Incidence, prevalence, and global burden of schizophrenia - data, with critical appraisal, from the Global Burden of Disease (GBD) 2019. Mol Psychiatry.

[3] GBD 2019 Mental Disorders Collaborators (2022). Global, regional, and national burden of 12 mental disorders in 204 countries and territories, 1990–2019: a systematic analysis for the Global Burden of Disease Study 2019. Lancet Psychiatry.

[4] Nolan KA, Volavka J, Czobor P (2005). Aggression and psychopathology in treatment-resistant inpatients with schizophrenia and schizoaffective disorder. J Psychiatr Res.

[5] Bagaric D, Brecic P, Ostojic D, Jukic V, Goles A (2013). The relationship between depressive syndrome and suicidal risk in patients with acute schizophrenia. Croat Med J.

[6] Fazel S, Gulati G, Linsell L, Geddes JR, Grann M (2009). Schizophrenia and violence: systematic review and meta-analysis. PLoS Med.

[7] Lamberti JS (2007). Understanding and preventing criminal recidivism among adults with psychotic disorders. Psychiatr Serv.

[8] Gleeson JF, McGuckian TB, Fernandez DK (2024). Systematic review of early warning signs of relapse and behavioural antecedents of symptom worsening in people living with schizophrenia spectrum disorders. Clin Psychol Rev.

[9] Sato A, Moriyama T, Watanabe N, Maruo K, Furukawa TA (2023). Development and validation of a prediction model for rehospitalization among people with schizophrenia discharged from acute inpatient care. Front Psychiatry.

[10] Cohen A, Naslund JA, Chang S (2023). Relapse prediction in schizophrenia with smartphone digital phenotyping during COVID-19: a prospective, three-site, two-country, longitudinal study. Schizophrenia (Heidelb).

[11] Intharit J, Kittiwattanagul K, Chaveepojnkamjorn W, Tudpor K (2021). Risk and protective factors of relapse in patients with first-episode schizophrenia from perspectives of health professionals: a qualitative study in northeastern Thailand. F1000Res.

[12] Gündoğmuş İ, Aydin MB, Öz S, Taşçi AB, Uzun Ö (2021). Clinical and demographic factors associated with early relapse in patients with schizophrenia: a naturalistic observation study. Int Clin Psychopharmacol.

[13] Zabihi Poursaadati M, Maarefvand M, Bolhari J (2023). Caregivers’ experiences and perspectives of factors associated with relapse in Iranian people living with schizophrenia: a qualitative study. Int J Soc Psychiatry.

[14] Maramis MM, Sofyan Almahdy M, Atika A, Bagus Jaya Lesmana C, Gerick Pantouw J (2022). The biopsychosocial-spiritual factors influencing relapse of patients with schizophrenia. Int J Soc Psychiatry.

[15] Robinson D, Woerner MG, Alvir JM (1999). Predictors of relapse following response from a first episode of schizophrenia or schizoaffective disorder. Arch Gen Psychiatry.

[16] Ren B, Yoon W, Thomas S, Savova G, Miller T, Hall MH (2025). Cross-site predictions of readmission after psychiatric hospitalization with mood or psychotic disorders: retrospective study. JMIR Ment Health.

[17] Fond G, Bulzacka E, Boucekine M (2019). Machine learning for predicting psychotic relapse at 2 years in schizophrenia in the national FACE-SZ cohort. Prog Neuropsychopharmacol Biol Psychiatry.

[18] Zhang L, Qi X, Wen L (2023). Identifying risk factors to predict violent behaviour in community patients with severe mental disorders: a retrospective study of 5277 patients in China. Asian J Psychiatr.

[19] Moges S, Belete T, Mekonen T, Menberu M (2021). Lifetime relapse and its associated factors among people with schizophrenia spectrum disorders who are on follow up at Comprehensive Specialized Hospitals in Amhara region, Ethiopia: a cross-sectional study. Int J Ment Health Syst.

[20] Olivares JM, Sermon J, Hemels M, Schreiner A (2013). Definitions and drivers of relapse in patients with schizophrenia: a systematic literature review. Ann Gen Psychiatry.

[21] Royston P (2004). Multiple imputation of missing values. Stata J.

[22] Nohara Y, Matsumoto K, Soejima H, Nakashima N (2022). Explanation of machine learning models using Shapley Additive Explanation and application for real data in hospital. Comput Methods Programs Biomed.

[23] van den Goorbergh R, van Smeden M, Timmerman D, Van Calster B (2022). The harm of class imbalance corrections for risk prediction models: illustration and simulation using logistic regression. J Am Med Inform Assoc.

[24] Kopelowicz A, Liberman RP, Zarate R (2006). Recent advances in social skills training for schizophrenia. Schizophr Bull.

[25] Fond G, Salas S, Pauly V (2019). End-of-life care among patients with schizophrenia and cancer: a population-based cohort study from the French national hospital database. Lancet Public Health.

[26] Florentin S, Neumark Y, Roe D (2024). The relationship between community-based psychiatric rehabilitation pathways and rehospitalization trajectories: a three-decade follow-up. Psychiatry Res.

[27] Robinson DG, Woerner MG, McMeniman M, Mendelowitz A, Bilder RM (2004). Symptomatic and functional recovery from a first episode of schizophrenia or schizoaffective disorder. Am J Psychiatry.

[28] Chen L, Zeng S, Su Y (2023). The influence of social exclusion on adolescents’ social withdrawal behavior: the moderating role of connectedness to nature. J Environ Psychol.

[29] Cornwell EY, Waite LJ (2009). Social disconnectedness, perceived isolation, and health among older adults. J Health Soc Behav.

[30] Georgiades A, Almuqrin A, Rubinic P, Mouhitzadeh K, Tognin S, Mechelli A (2023). Psychosocial stress, interpersonal sensitivity, and social withdrawal in clinical high risk for psychosis: a systematic review. Schizophrenia (Heidelb).

[31] Marks JR, Schectman JM, Groninger H, Plews-Ogan ML (2010). The association of health literacy and socio-demographic factors with medication knowledge. Patient Educ Couns.

[32] Agenagnew L, kassaw C (2020). The lifetime prevalence and factors associated with relapse among mentally ill patients at Jimma University Medical Center, Ethiopia: cross sectional study. J Psychosoc Rehabil Ment Health.

[33] Ascher-Svanum H, Zhu B, Faries DE (2010). The cost of relapse and the predictors of relapse in the treatment of schizophrenia. BMC Psychiatry.

