# Supplementary material for: Analysis and Prediction of Relapse Risk Factors in Community-Dwelling Individuals With Schizophrenia: Retrospective Cohort Study
Source: JMIR Med Inform. 2026 Oct 28;14:e86991. doi: 10.2196/86991 (PMC13716292; doi:10.2196/86991)
Supplement: Supplementary file 1 [file medinform-v14-e86991-s001.docx]

Appendix file 1 Baseline characteristics, variable coding, and statistical comparisons by relapse status.

Table 1-5 five thematic domains

Categorical variables were numerically encoded in ascending order according to the category sequence presented in the table (e.g., Male = 0, Female = 1).

Table 1 Sociodemographic Characteristics

| **Variables** | **Non-relapse** | **Relapse** | **Total** | **Z/X^2^** | **P** |
| --- | --- | --- | --- | --- | --- |
| Age | 61(47,69) | 54(42,64) | 60(47,69) | -20.78 | <.001 |
| Number of Follow-ups | 5(3,7) | 5(2,10) | 5(3,8) | -0.06 | 0.95 |
| Sex |  |  |  | 48.25 | <.001 |
| Male | 16706(46.3%) | 1942(52.2%) | 18648(46.8%) |  |  |
| Female | 19395(53.7%) | 1775(47.8%) | 21170(53.2%) |  |  |
| Educational Attainment |  |  |  | 98.73 | <.001 |
| Primary School or Illiterate/Semi-illiterate | 4012(11.1%) | 264(7.1%) | 4276(10.7%) |  |  |
| Junior High School / Senior High School | 25763(71.4%) | 2614(70.3%) | 28377(71.3%) |  |  |
| Junior College/Bachelor's Degree/Master's Degree or above | 6326(17.5%) | 839(22.6%) | 7165(18%) |  |  |
| Marital Status |  |  |  | 339.34 | <.001 |
| Unmarried | 15234(42.2%) | 2054(55.3%) | 17288(43.4%) |  |  |
| Married | 16488(45.7%) | 1114(30%) | 17602(44.2%) |  |  |
| Other Marital Status | 4379(12.1%) | 549(14.8%) | 4928(12.4%) |  |  |
| Long-term Bedridden Status or Loss of Mobility |  |  |  | 0.11 | 0.74 |
| No | 35105(97.2%) | 3611(97.1%) | 38716(97.2%) |  |  |
| Yes | 996(2.8%) | 106(2.9%) | 1102(2.8%) |  |  |
| Family Supervision Status | **Non-relapse** | **Relapse** | **Total** | 276.73 | <.001 |
| No Supervision | 199(0.6%) | 40(1.1%) | 239(0.6%) |  |  |
| Weak | 4076(11.3%) | 755(20.3%) | 4831(12.1%) |  |  |
| Good | 31826(88.2%) | 2922(78.6%) | 34748(87.3%) |  |  |
| Participation in Community Rehabilitation Services |  |  |  | 0.23 | 0.63 |
| No | 2919(8.1%) | 309(8.3%) | 3228(8.1%) |  |  |
| Yes | 33182(91.9%) | 3408(91.7%) | 36590(91.9%) |  |  |

Table 2 Clinical Diagnosis and Medical History

| **Variables** | **Non-relapse** | **Relapse** | **Total** | **Z/X^2^** | **P** |
| --- | --- | --- | --- | --- | --- |
| Antipsychotic Treatment Status |  |  |  | 2.01 | 0.16 |
| No | 269(0.7%) | 20(0.5%) | 289(0.7%) |  |  |
| Yes | 35832(99.3%) | 3697(99.5%) | 39529(99.3%) |  |  |
| Reason for Untreated Status |  |  |  | 71.54 | <.001 |
| None | 33539(92.9%) | 3580(96.3%) | 37119(93.2%) |  |  |
| Financial Constraints | 1489(4.1%) | 75(2%) | 1564(3.9%) |  |  |
| Perceived Recovery | 100(0.3%) | 14(0.4%) | 114(0.3%) |  |  |
| Lack of Confidence in Treatment | 10(0%) | 2(0.1%) | 12(0%) |  |  |
| Adverse Drug Reactions | 5(0%) | 0(0%) | 5(0%) |  |  |
| Other Reasons | 958(2.7%) | 46(1.2%) | 1004(2.5%) |  |  |
| Recent Stressful Events |  |  |  | 70.75 | <.001 |
| None | 35962(99.6%) | 3667(98.7%) | 39629(99.5%) |  |  |
| Mild | 133(0.4%) | 45(1.2%) | 178(0.4%) |  |  |
| Severe | 6(0%) | 5(0.1%) | 11(0%) |  |  |
| Social Functioning Assessment |  |  |  | 1370.99 | <.001 |
| Poor | 7914(21.9%) | 1785(48%) | 9699(24.4%) |  |  |
| Moderate | 21886(60.6%) | 1748(47%) | 23634(59.4%) |  |  |
| Good | 6301(17.5%) | 184(5%) | 6485(16.3%) |  |  |
| Insight into Illness |  |  |  | 1999.39 | <.001 |
| Absent Insight | 5039(14%) | 1557(41.9%) | 6596(16.6%) |  |  |
| Partial Insight | 23334(64.6%) | 1862(50.1%) | 25196(63.3%) |  |  |
| Full Insight | 7728(21.4%) | 298(8%) | 8026(20.2%) |  |  |
| History of Violent or Disruptive Behavior |  |  |  | 329.88 | <.001 |
| No | 35634(98.7%) | 3520 (94.7%) | 39154 (98.3%) |  |  |
| Yes | 467 (1.3%) | 197 (5.3%) | 664 (1.7%) |  |  |
| Mode of Illness Onset |  |  |  | 2.56 | 0.28 |
| Insidious Onset | 17881(49.5%) | 1888(50.8%) | 19769(49.6%) |  |  |
| Subacute Onset | 11337(31.4%) | 1124(30.2%) | 12461(31.3%) |  |  |
| Acute Onset | 6883(19.1%) | 705(19%) | 7588(19.1%) |  |  |
| Past Medical History |  |  |  | 8.20 | 0.004 |
| No History | 32546(90.2%) | 3296(88.7%) | 35842(90%) |  |  |
| Positive History | 3555(9.8%) | 421(11.3%) | 3976(10%) |  |  |
| Disability Severity Level |  |  |  | 162.03 | <.001 |
| No Disability | 19010(52.7%) | 1579(42.5%) | 20589(51.7%) |  |  |
| Level I Disability | 4196(11.6%) | 507(13.6%) | 4703(11.8%) |  |  |
| Level II Disability | 9468(26.2%) | 1261(33.9%) | 10729(26.9%) |  |  |
| Level III Disability | 1740(4.8%) | 216(5.8%) | 1956(4.9%) |  |  |
| Level IV Disability | 1687(4.7%) | 154(4.1%) | 1841(4.6%) |  |  |

Table 3 Medication Management and Follow-up

| **Variables** | **Non-relapse** | **Relapse** | **Total** | **Z/X^2^** | **P** |
| --- | --- | --- | --- | --- | --- |
| Medication Persistence |  |  |  | 108.20 | <.001 |
| Refusal of Medication | 2675(7.4%) | 147(4%) | 2822(7.1%) |  |  |
| Intermittent Medication Use | 1169(3.2%) | 63(1.7%) | 1232(3.1%) |  |  |
| Continuous Medication Use | 31833(88.2%) | 3488(93.8%) | 35321(88.7%) |  |  |
| Medication Not Required per Medical Advice | 424(1.2%) | 19(0.5%) | 443(1.1%) |  |  |
| Medication Administration Pattern |  |  |  | 1376.48 | <.001 |
| Refusal of Medication | 2675(7.4%) | 147(4%) | 2822(7.1%) |  |  |
| Medication Administered by Caregiver | 15292(42.4%) | 2756(74.1%) | 18048(45.3%) |  |  |
| Self-administered Medication | 17709(49.1%) | 794(21.4%) | 18503(46.5%) |  |  |
| Medication Not Required per Medical Advice | 425(1.2%) | 20(0.5%) | 445(1.1%) |  |  |
| Medication Adherence |  |  |  | 93.37 | <.001 |
| Refusal of Medication | 2675(7.4%) | 147(4%) | 2822(7.1%) |  |  |
| Irregular Medication Adherence | 924(2.6%) | 57(1.5%) | 981(2.5%) |  |  |
| Regular Medication Adherence | 32077(88.9%) | 3493(94%) | 35570(89.3%) |  |  |
| Medication Not Required per Medical Advice | 425(1.2%) | 20(0.5%) | 445(1.1%) |  |  |
| Timeliness of Clinical Follow-up |  |  |  | 657.18 | <.001 |
| No Outpatient Follow-up | 8103(22.4%) | 1536(41.3%) | 9639(24.2%) |  |  |
| Irregular Outpatient Visits | 2351(6.5%) | 159(4.3%) | 2510(6.3%) |  |  |
| Regular Outpatient Visits | 25647(71%) | 2022(54.4%) | 27669(69.5%) |  |  |

Table 4 Rehabilitation Participation

| **Variables** | **Non-relapse** | **Relapse** | **Total** | **Z/X^2^** | **P** |
| --- | --- | --- | --- | --- | --- |
| Social Participation |  |  |  | 1998.39 | <.001 |
| No Social Participation | 6688(18.5%) | 1836(49.4%) | 8524(21.4%) |  |  |
| Temporary Employment | 773(2.1%) | 33(0.9%) | 806(2%) |  |  |
| Basic Household Activities | 22910(63.5%) | 1640(44.1%) | 24550(61.7%) |  |  |
| Participation in Public Activities | 2271(6.3%) | 144(3.9%) | 2415(6.1%) |  |  |
| Regular Employment Participation | 3459(9.6%) | 64(1.7%) | 3523(8.8%) |  |  |
| Sleep Quality |  |  |  | 1866.47 | <.001 |
| Poor | 4717(13.1%) | 1488(40%) | 6205(15.6%) |  |  |
| Moderate | 21481(59.5%) | 1576(42.4%) | 23057(57.9%) |  |  |
| Good | 9903(27.4%) | 653(17.6%) | 10556(26.5%) |  |  |
| Dietary Status |  |  |  | 2220.89 | <.001 |
| Poor | 3881(10.8%) | 1424(38.3%) | 5305(13.3%) |  |  |
| Moderate | 20191(55.9%) | 1495(40.2%) | 21686(54.5%) |  |  |
| Good | 12029(33.3%) | 798(21.5%) | 12827(32.2%) |  |  |
| Personal Self-care Ability |  |  |  | 1878.04 | <.001 |
| Poor | 5123(14.2%) | 1548(41.6%) | 6671(16.8%) |  |  |
| Moderate | 19963(55.3%) | 1601(43.1%) | 21564(54.2%) |  |  |
| Good | 11015(30.5%) | 568(15.3%) | 11583(29.1%) |  |  |
| Household Functioning |  |  |  | 1704.46 | <.001 |
| Poor | 6167(17.1%) | 1666(44.8%) | 7833(19.7%) |  |  |
| Moderate | 21074(58.4%) | 1645(44.3%) | 22719(57.1%) |  |  |
| Good | 8860(24.5%) | 406(10.9%) | 9266(23.3%) |  |  |
| Occupational Functioning and Productive Work |  |  |  | 1640.81 | <.001 |
| Good | 3132(8.7%) | 77(2.1%) | 3209(8.1%) |  |  |
| Moderate | 11094(30.7%) | 738(19.9%) | 11832(29.7%) |  |  |
| Poor | 6451(17.9%) | 1685(45.3%) | 8136(20.4%) |  |  |
| No Work | 15424(42.7%) | 1217(32.7%) | 16641(41.8%) |  |  |
| Acquisition of New Knowledge and Skills |  |  |  | 851.45 | <.001 |
| Poor | 11119(30.8%) | 1999(53.8%) | 13118(32.9%) |  |  |
| Moderate | 21224(58.8%) | 1581(42.5%) | 22805(57.3%) |  |  |
| Good | 3758(10.4%) | 137(3.7%) | 3895(9.8%) |  |  |
| Interpersonal Social Interaction |  |  |  | 1218.51 | <.001 |
| Poor | 9904(27.4%) | 2016(54.2%) | 11920(29.9%) |  |  |
| Moderate | 22030(61%) | 1580(42.5%) | 23610(59.3%) |  |  |
| Good | 4167(11.5%) | 121(3.3%) | 4288(10.8%) |  |  |

Table 5 Rehabilitation Interventions and Specialist Recommendations

| **Variables** | **Non-relapse** | **Relapse** | **Total** | **Z/X^2^** | **P** |
| --- | --- | --- | --- | --- | --- |
| Rehabilitation Intervention (Daily Living and Vocational Skills) |  |  |  | 0.04 | 0.85 |
| Not Required | 14686(40.7%) | 1518(40.8%) | 16204(40.7%) |  |  |
| Required | 21415(59.3%) | 2199(59.2%) | 23614(59.3%) |  |  |
| Rehabilitation Intervention (Vocational Training) |  |  |  | 6.95 | 0.008 |
| Not Required | 34193(94.7%) | 3558(95.7%) | 37751(94.8%) |  |  |
| Required | 1908(5.3%) | 159(4.3%) | 2067(5.2%) |  |  |
| Rehabilitation Intervention (Learning Ability) |  |  |  | 22.84 | <.001 |
| Not Required | 32796(90.8%) | 3464(93.2%) | 36260(91.1%) |  |  |
| Required | 3305(9.2%) | 253(6.8%) | 3558(8.9%) |  |  |
| Rehabilitation Intervention (Social Interaction) |  |  |  | 188.03 | <.001 |
| Not Required | 23976(66.4%) | 2880(77.5%) | 26856(67.4%) |  |  |
| Required | 12125(33.6%) | 837(22.5%) | 12962(32.6%) |  |  |
| Other Rehabilitation Interventions |  |  |  | 187.23 | <.001 |
| Not Required | 29837(82.6%) | 2734(73.6%) | 32571(81.8%) |  |  |
| Required | 6264(17.4%) | 983(26.4%) | 7247(18.2%) |  |  |
| Specialist Recommendation: Treatment Adjustment |  |  |  | 3207.36 | <.001 |
| Not Required | 35257(97.7%) | 2991(80.5%) | 38248(96.1%) |  |  |
| Maintain Current Treatment | 423(1.2%) | 119(3.2%) | 542(1.4%) |  |  |
| Medication Adjustment | 421(1.2%) | 607(16.3%) | 1028(2.6%) |  |  |
| Specialist Recommendation: Vocational Rehabilitation Training |  |  |  | 1.18 | 0.28 |
| Not Required | 35386(98%) | 3653(98.3%) | 39039(98%) |  |  |
| Required | 715(2%) | 64(1.7%) | 779(2%) |  |  |
| Specialist Recommendation: Symptom Monitoring Training |  |  |  | 2.36 | 0.13 |
| Not Required | 28650(79.4%) | 2910(78.3%) | 31560(79.3%) |  |  |
| Required | 7451(20.6%) | 807(21.7%) | 8258(20.7%) |  |  |
| Specialist Recommendation: Learning Skills Training |  |  |  | 19.91 | <.001 |
| Not Required | 34348(95.1%) | 3597(96.8%) | 37945(95.3%) |  |  |
| Required | 1753(4.9%) | 120(3.2%) | 1873(4.7%) |  |  |
| Specialist Recommendation: Social Skills Training |  |  |  | 116.65 | <.001 |
| Not Required | 30399(84.2%) | 3378(90.9%) | 33777(84.8%) |  |  |
| Required | 5702(15.8%) | 339(9.1%) | 6041(15.2%) |  |  |
| Specialist Recommendation: Daily Living Skills Training |  |  |  | 232.46 | <.001 |
| Not Required | 23991(66.5%) | 2927(78.7%) | 26918(67.6%) |  |  |
| Required | 12110(33.5%) | 790(21.3%) | 12900(32.4%) |  |  |
| Specialist Recommendation: Medication Adherence Training |  |  |  | 670.32 | <.001 |
| Not Required | 26273(72.8%) | 1952(52.5%) | 28225(70.9%) |  |  |
| Required | 9828(27.2%) | 1765(47.5%) | 11593(29.1%) |  |  |

Table 6 Normalized mean absolute SHAP importance values for all 41 modeled features.

|  | **Feature** | **Mean_Abs_SHAP** | **Importance_Pct** |
| --- | --- | --- | --- |
| 1 | Medication Persistence | 0.94 | 23.3 |
| 2 | Specialist Recommendation: Medication Adherence Training | 0.79 | 19.74 |
| 3 | Number of Follow-ups | 0.30 | 7.38 |
| 4 | Age | 0.15 | 3.72 |
| 5 | Educational Attainment | 0.14 | 3.58 |
| 6 | Occupational Functioning and Productive Work | 0.13 | 3.11 |
| 7 | Family Supervision Status | 0.12 | 2.94 |
| 8 | Medication Administration Pattern | 0.12 | 2.9 |
| 9 | Social Functioning Assessment | 0.11 | 2.8 |
| 10 | Specialist Recommendation: Treatment Adjustment | 0.09 | 2.27 |
| 11 | Disability Severity Level | 0.09 | 2.21 |
| 12 | Medication Adherence | 0.09 | 2.21 |
| 13 | Social Participation | 0.09 | 2.15 |
| 14 | Acquisition of New Knowledge and Skills | 0.07 | 1.76 |
| 15 | Sex | 0.07 | 1.66 |
| 16 | Timeliness of Clinical Follow-up | 0.06 | 1.58 |
| 17 | Recent Stressful Events | 0.06 | 1.56 |
| 18 | Household Functioning | 0.06 | 1.46 |
| 19 | Specialist Recommendation: Daily Living Skills Training | 0.06 | 1.42 |
| 20 | Rehabilitation Intervention (Social Interaction) | 0.04 | 1.05 |
| 21 | Interpersonal Social Interaction | 0.04 | 1 |
| 22 | Sleep Quality | 0.04 | 0.93 |
| 23 | Marital Status | 0.03 | 0.84 |
| 24 | Past Medical History | 0.03 | 0.83 |
| 25 | Insight into Illness | 0.03 | 0.79 |
| 26 | Specialist Recommendation: Social Skills Training | 0.03 | 0.78 |
| 27 | Long-term Bedridden Status or Loss of Mobility | 0.03 | 0.77 |
| 28 | Dietary Status | 0.03 | 0.69 |
| 29 | Specialist Recommendation: Symptom Monitoring Training | 0.03 | 0.63 |
| 30 | Other Rehabilitation Interventions | 0.03 | 0.63 |
| 31 | Personal Self-care Ability | 0.02 | 0.6 |
| 32 | Mode of Illness Onset | 0.02 | 0.51 |
| 33 | History of Violent or Disruptive Behavior | 0.02 | 0.47 |
| 34 | Participation in Community Rehabilitation Services | 0.022 | 0.46 |
| 35 | Rehabilitation Intervention (Daily Living and Vocational Skills) | 0.02 | 0.44 |
| 36 | Rehabilitation Intervention (Learning Ability) | 0.01 | 0.26 |
| 37 | Reason for Untreated Status | 0.01 | 0.23 |
| 38 | Specialist Recommendation: Learning Skills Training | 0.01 | 0.16 |
| 39 | Rehabilitation Intervention (Vocational Training) | 0.00 | 0.1 |
| 40 | Specialist Recommendation: Vocational Rehabilitation Training | 0.00 | 0.07 |
| 41 | Antipsychotic Treatment Status | 0.00 | 0.03 |
