# Supplementary material for: Analysis and Prediction of Relapse Risk Factors in Community-Dwelling Individuals With Schizophrenia: Retrospective Cohort Study
Source: JMIR Med Inform. 2026 Oct 28;14:e86991. doi: 10.2196/86991 (PMC13716292; doi:10.2196/86991)
Supplement: Supplementary file 2 [file medinform-v14-e86991-s002.docx]

Appendix File 2 Detailed machine learning model development, hyperparameter optimization, model validation, sensitivity analyses, and SHAP feature importance.

**Machine Learning Algorithms**

To comprehensively evaluate predictive performance and model robustness, six commonly used machine learning algorithms were implemented and compared in this study:

Light Gradient Boosting Machine (LightGBM)

Random Forest (RF)

Adaptive Boosting (AdaBoost)

Support Vector Machine (SVM)

Logistic Regression (LR)

Extreme Gradient Boosting (XGBoost)

All models were implemented using Python 3.10 with the scikit-learn, lightgbm, and xgboost libraries.

**Data Splitting Strategy**

The dataset was divided into training and independent testing subsets using stratified sampling to preserve the original relapse/non-relapse distribution.

Training set: 80%

Independent test set: 20%

The independent test set maintained the original class distribution and was not modified during model development. To prevent information leakage, all preprocessing procedures, including missing-value handling, feature selection, feature scaling where applicable, class-weight parameter optimization, and hyperparameter tuning, were performed exclusively within the training set. The final model performance was evaluated only on the held-out independent test set.

**Handling Class Imbalance**

Because the original dataset exhibited substantial class imbalance, cost-sensitive learning strategies were applied to improve recognition of hospitalization-related severe relapse cases without changing the original sample distribution.

Specifically, LightGBM and XGBoost used the scale_pos_weight parameter; Random Forest, SVM, and Logistic Regression used the class_weight parameter; and AdaBoost used class-dependent sample weights. All weighting parameters were incorporated into the grid-search procedure and optimized through five-fold stratified cross-validation.

**Hyperparameter Optimization**

Hyperparameter optimization was conducted using GridSearchCV combined with 5-fold stratified cross-validation, with ROC-AUC serving as the primary optimization metric. Class imbalance was addressed through cost-sensitive learning strategies rather than resampling. Specifically, scale_pos_weight was applied in boosting-based models (LightGBM and XGBoost), class_weight was used in Random Forest, Support Vector Machine (SVM), and Logistic Regression, and sample_weight was adopted in AdaBoost. No oversampling or undersampling techniques were applied. The hyperparameter search spaces and the final optimal configurations for each model are presented below.

***LightGBM***

Search space:

learning_rate ∈ {0.05, 0.1, 0.2}

max_depth ∈ {5, 8, 13}

num_leaves ∈ {31, 63, 127}

n_estimators ∈ {100, 200, 300}

scale_pos_weight ∈ {1, 3, 5, 10}

Optimal parameters:

learning_rate = 0.1

max_depth = 13

num_leaves = 31

n_estimators = 100

scale_pos_weight = 3

feature_fraction=0.9

subsample=0.67

reg_alpha=0.4

reg_lambda=0.3.

These parameters were not included in the grid search. subsample_freq remained at its default value of 0, so row subsampling was disabled.

***Random Forest***

Search space:

n_estimators ∈ {100, 200, 300}

max_depth ∈ {5, 10, 15}

min_samples_split ∈ {2, 5, 10}

class_weight ∈ {balanced, balanced_subsample}

Optimal parameters:

n_estimators = 300

max_depth = 15

min_samples_split = 10

class_weight = balanced

***AdaBoost***

Search space:

n_estimators ∈ {50, 100, 200}

learning_rate ∈ {0.01, 0.1, 0.2}

Optimal parameters:

n_estimators = 200

learning_rate = 0.2

As AdaBoost does not support the class_weight parameter, cost-sensitive learning was achieved by assigning higher sample weights (sample_weight) to relapse cases, thereby increasing the model's attention to the minority class during training.

***SVM***

Search space:

C ∈ {0.1, 1, 10}

gamma ∈ {scale, auto, 0.1}

kernel = rbf

class_weight ∈ {balanced, None}

Optimal parameters:

C = 1

gamma = auto

kernel = rbf

class_weight = balanced

Since SVM is sensitive to differences in feature scale, all input features were standardized using StandardScaler before model training. To avoid data leakage, the scaler was fitted only on the training set and then applied to the corresponding test set.

***Logistic Regression***

Search space:

C ∈ {0.1, 1, 10}

penalty ∈ {l1, l2}

solver = liblinear

class_weight ∈ {balanced, None}

Optimal parameters:

C = 0.1

penalty = l2

solver = liblinear

class_weight = balanced

The features were also standardized before model training.

***XGBoost***

Search space:

n_estimators ∈ {100, 200, 300}

learning_rate ∈ {0.05, 0.1, 0.2}

max_depth ∈ {3, 5, 7}

subsample ∈ {0.8, 1}

scale_pos_weight ∈ {1, 3, 5, 10}

Optimal parameters:

n_estimators = 300

learning_rate = 0.1

max_depth = 5

subsample = 0.8

scale_pos_weight = 3

**Feature Scaling**

Feature standardization was applied only to algorithms sensitive to variable scales:

SVM and Logistic Regression

Tree-based models (LightGBM, Random Forest, AdaBoost, and XGBoost) were trained using the original feature scales without normalization.

**Model Evaluation**

Model performance was evaluated on the independent test set using the following metrics:

Accuracy

Precision

Recall (Sensitivity)

F1-score

ROC-AUC

Macro-averaged metrics

Confusion matrices were additionally generated to assess relapse-specific classification performance.

**Reproducibility**

To support reproducibility within the restricted analysis environment, all random number generators were initialized using random_state = 42.

**Probability Calibration**

Calibration performance was evaluated using the retained random 20% test set. Because the primary LightGBM model was optimized for relapse classification using cost-sensitive learning (scale_pos_weight = 3), we additionally evaluated an unweighted baseline model (scale_pos_weight = 1) to examine probability calibration under the same training pipeline. The unweighted baseline model achieved a Brier score of 0.036, and its calibration curve demonstrated good agreement between predicted probabilities and observed relapse frequencies. In comparison, the primary cost-sensitive classification model yielded a slightly higher Brier score of 0.040. This difference reflects the different optimization objectives of the two models. Cost-sensitive learning improves minority-class recall by assigning greater weight to relapse cases during model training, but it may also shift predicted probabilities toward the positive class, resulting in a modest reduction in probability calibration. In other words, a trade-off may exist between calibration and minority-class recall in cost-sensitive classification.

Accordingly, we used the unweighted baseline model to evaluate and report calibration performance, as its probability estimates were not affected by class-weight adjustment, whereas the cost-sensitive model was retained for the primary classification task. In practical deployment, model outputs should be interpreted in conjunction with application-specific decision thresholds. Alternatively, post hoc recalibration methods, such as Platt scaling or isotonic regression, may be applied to the predicted probabilities to improve calibration while preserving the recall advantage of the cost-sensitive model.

Table 1 Calibrate Brier score

| Model | Brier Score | Purpose |
| --- | --- | --- |
| LightGBM (scale_pos_weight = 1, unweighted baseline model) | 0.036 | Calibration assessment (probability estimates not affected by cost-sensitive weighting) |
| LightGBM (scale_pos_weight = 3, cost-sensitive model) | 0.040 | Primary classification model (higher recall with shifted probability estimates) |

Figure 1 Calibration Curve (LightGBM)

**Confidence Intervals**

In the random 80/20 test set used in the primary analysis (n=7,964; relapse cases, n=743), the 95% confidence intervals for the performance metrics of the primary LightGBM model were estimated using stratified bootstrap resampling (B=2,000). The 95% confidence interval for ROC-AUC was additionally validated using the analytical DeLong method. The intervals were narrow, indicating that the reported estimates were precise and stable.

Table 2. Point estimates and 95% confidence intervals for the performance metrics of the primary LightGBM model.

| Metric | Point Estimate | 95% CI (Bootstrap) | Validation |
| --- | --- | --- | --- |
| ROC-AUC | 0.95 | [0.95, 0.96] | DeLong [0.95, 0.96] |
| PR-AUC | 0.79 | [0.77, 0.82] | — |
| Relapse Recall | 0.77 | [0.74, 0.80] | — |
| Relapse Precision | 0.71 | [0.68, 0.73] | — |
| Relapse F1 | 0.74 | [0.71, 0.76] | — |
| Accuracy | 0.95 | [0.94, 0.95] | — |

Figure 2 Point estimates and 95% confidence intervals for six performance metrics of the primary LightGBM model (forest plot)

**Exploratory Temporal Robustness Check**

An exploratory temporal robustness check was conducted according to the calendar year of the retained patient-level index record. Using the same modeling pipeline as in the primary random 80/20 split analysis, the LightGBM model was trained on the 2022 index-record cohort and evaluated on the 2023 index-record cohort.

The F1-maximizing operating threshold was selected using in-sample predictions from the 2022 cohort used to fit the model; therefore, the F1 value used for threshold selection may be optimistic, although the threshold was fixed before evaluation in the 2023 cohort.

The 2022 index-record cohort included 9,028 patients, with a hospitalization-related severe relapse prevalence of approximately 19.7%. The 2023 index-record cohort included 30,790 patients, with a relapse prevalence of 6.3%. Among the 30,790 patients in the 2023 cohort, 21,566 had retained index records in the fourth quarter of 2023. Because the calendar year of the retained index record was partly influenced by the index-record selection rule, this analysis should be interpreted as an exploratory robustness check rather than as a conventional independent temporal validation.

Table 3. Quarter-wise discrimination performance and relapse prevalence in the 2023 index-record cohort, with the model trained on the 2022 index-record cohort.

| Period | n | Relapse Prevalence | ROC-AUC | 95% CI | PR-AUC |
| --- | --- | --- | --- | --- | --- |
| 2023 Q1 | 954 | 31.0% | 0.99 | [0.98, 0.99] | 0.97 |
| 2023 Q2 | 2118 | 13.7% | 0.98 | [0.97, 0.99] | 0.89 |
| 2023 Q3 | 6152 | 5.5% | 0.95 | [0.94, 0.97] | 0.74 |
| 2023 Q4 | 21566 | 4.7% | 0.88 | [0.87, 0.89] | 0.36 |
| Overall 2023 | 30790 | 6.3% | 0.93 | [0.92, 0.93] | 0.62 |

Quarter-wise results in the 2023 index-record cohort showed that ROC-AUC remained above 0.88 across all four quarters, whereas PR-AUC decreased as relapse prevalence declined from 31.0% in Q1 to 4.7% in Q4. This pattern suggests that changes in relapse prevalence, case mix, and the index-record selection rule likely contributed to the decline in PR-AUC and operating-point performance. The lower PR-AUC observed in Q4 further indicates the need for continued performance monitoring and potential recalibration before long-term deployment.

Figure 3. Quarter-wise discrimination performance in the 2023 index-record cohort.

Figure 4. Decision curve analysis in the 2023 index-record cohort comparing the LightGBM model with the treat-all and treat-none strategies.

**Sensitivity Analysis**

To evaluate whether the performance of the model was overly dependent on management-related or potentially leakage-prone variables, we conducted a stepwise sensitivity analysis by sequentially removing related variables from the model.

The variables removed in this analysis included: (1) Number of Follow-ups; (2) Specialist Recommendation: Treatment Adjustment; (3) Specialist Recommendation: Medication Adherence Training; (4) Timeliness of Clinical Follow-up; (5) Medication Persistence; (6) Medication Administration Pattern; and (7) Medication Adherence. After each removal step, the LightGBM model was retrained and evaluated using the same random 80/20 data split and a fixed classification threshold of 0.5.

Table 4. Model performance after stepwise removal of related variable clusters using LightGBM in the random 80/20 test set at a threshold of 0.5.

| Feature Set | ROC-AUC | PR-AUC | Relapse Recall | Relapse Precision | Relapse F1 | ΔROC vs FULL | ΔPR vs FULL |
| --- | --- | --- | --- | --- | --- | --- | --- |
| FULL | 0.95 | 0.79 | 0.77 | 0.71 | 0.74 | — | — |
| REDUCED-3^a^ | 0.92 | 0.74 | 0.70 | 0.72 | 0.71 | −0.03 | −0.05 |
| REDUCED-6^b^ | 0.92 | 0.75 | 0.62 | 0.83 | 0.71 | −0.03 | −0.04 |
| REDUCED-7^c^ | 0.92 | 0.75 | 0.62 | 0.82 | 0.71 | −0.03 | −0.04 |

a REDUCED-3 (removal of the 3-variable management-related cluster: Number of Follow-ups, Specialist Recommendation: Treatment Adjustment, and Specialist Recommendation: Medication Adherence Training)

b REDUCED-6 (removal of the 6-variable management-related cluster: Number of Follow-ups, Specialist Recommendation: Treatment Adjustment, Specialist Recommendation: Medication Adherence Training, Timeliness of Clinical Follow-up, Medication Persistence, and Medication Administration Pattern)

c REDUCED-7 (removal of the 7-variable management-related cluster: Number of Follow-ups, Specialist Recommendation: Treatment Adjustment, Specialist Recommendation: Medication Adherence Training, Timeliness of Clinical Follow-up, Medication Persistence, Medication Administration Pattern, and Medication Adherence)

**Encoding sensitivity analysis**

Table 5. Encoding sensitivity analysis for SVM and LR

| Model | Coding | ROC-AUC | PR-AUC | Relapse Recall | Relapse Precision | Relapse F1 |
| --- | --- | --- | --- | --- | --- | --- |
| SVM | Integer encoding (ordinal) | 0.94 | 0.68 | 0.82 | 0.56 | 0.67 |
| SVM | One-hot encoding | 0.94 | 0.67 | 0.83 | 0.57 | 0.67 |
| Logistic Regression | Integer encoding (ordinal) | 0.85 | 0.40 | 0.78 | 0.26 | 0.39 |
| Logistic Regression | One-hot encoding | 0.85 | 0.40 | 0.78 | 0.26 | 0.39 |
